# Participatory epidemiology on major camel calf health and management problems in pastoral and semi-pastoral zones of Somali region, Ethiopia

**DOI:** 10.1371/journal.pone.0301551

**Published:** 2024-03-29

**Authors:** Hassan Abdi Hussein, Abdifetah Mohamed, Juhar Mohamed Ahmed

**Affiliations:** College of Veterinary Medicine, Jigjiga University, Jigjiga, Somali Region, Ethiopia; University of Naples Federico II: Universita degli Studi di Napoli Federico II, ITALY

## Abstract

A cross-sectional study was conducted from May 2017 to March 2019. A participatory epidemiological appraisal was used to gain a rapid overview of the range of camel calf health problems and traditional management, a measure of the importance that people place on each of them, and to identify and prioritize economically important diseases in target zones. The most important constraints to camel production were identified to be the widespread prevalence of diseases such as camelpox, contagious ecthyma, calf scour, ticks, and nonspecific pneumonia; poor management and husbandry practices such as restrictive colostrum feeding, lack of concentrate and salt supplementation and inappropriate housing; shortage of feed; and scarce seasonal variation in water. Additionally, the livestock herders not only showed their knowledge of common camel calf diseases for affected organs and symptoms but also indicated the seasonality of disease occurrences with strong agreement (W = 0.899, P< 0.003) among the informants of all focus group discussions. The overall prevalence of mange, tick infestation, and bacteria-induced diarrhea in the study area was found to be 36.3%, 36%, and 74%, respectively. *Sarcoptes scabie var*. *cameli* was the only identified mite species from mange-infested calves, while *Hyalomma*, *Rhipicephalus*, and *Amblyoma* were the most commonly identified tick species. Similarly, the overall prevalence of diarrhea was 74% among this about 34.6% was caused by *E*. *coli* while 38.9% was affected by *Salmonella* and *E*. *coli*. Therefore, based on these findings, five diseases have been prioritized as the most significant calf diseases in the area (Camelpox, contagious ecthyma, and causes of pneumonia among camel calves). Improving veterinary health infrastructure and capacity, and increasing community awareness on camel health constraints are also recommended to enhance optimal camel calf rearing.

## Introduction

In Ethiopia, camels (*Camelus dromedarius*) are a subset of the vast livestock resources that ensure the livelihood survival of an estimated ten million people who live in the arid and semiarid regions of the country, which make up more than 60% of the country’s total area and are home to pastoral and agro-pastoral communities [1]. Camel rearing is also the most sustainable livestock production system, as camel species are well adapted to hot and arid environments for pastoralists [2].

In the Somali region, camel rearing serves as an essential means of adapting human life to harsh and unfavorable environments. It proves to be an effective method of utilizing arid areas, where cultivating crops and engaging in small-scale cattle production are not feasible. Given the challenges posed by global warming and climate change, the significance of camels in sustaining pastoralists in eastern Ethiopia will grow even more vital [3]. However, camels are slow producers; the average gestation period is 14 months, often longer, and the prevalence of several diseases and climate change substantially hinder camel productivity. Excessive calf mortality is one of the main obstacles to improving camel productivity [4]. Since calves are replacement stock, the herd can only grow with them, and the death of calves will prevent camel producers from having access to milk. As a result, raising camel calves under traditional pastoral production systems presents several difficulties that lead to high mortality rates [5].

Studies indicate that diseases and starvation frequently cause young camel calves to die before they are eight months old [6]. The crude camel calf mortality rate reported in Ethiopia was 30% [7]. It is also estimated that camel calf losses could reach 45.5%, significantly impacting the herd’s replacement stock [8]. Whereas newborn camels are normally born with very little natural protection against diseases, they can draw immediate passive immune protection from maternal colostrum (given at the right time and in sufficient quantity) containing a high concentration of antibodies, complement and other immune elements [9]. Withholding of colostrum from neonates due to a belief among many pastoralists that it causes diarrhea is an important constraint contributing to high calf mortality in pastoral systems [10]. Several factors, including management, environmental, and animal factors may account for such a high death rate and deserve an in-depth investigation. Vast arrays of infectious diseases hampering adult camels’ health also affect camel calves [11].

In the Somali region, few investigations have been conducted on the assessment of young stock mortality causes including camel neonates but major camel calf diseases and their magnitude in camel-producing areas of the region particularly in pastoral and agro-pastoral areas where camel rearing is the mainstay are not sufficiently studied. The impact of diseases on camel calves is currently at an alarming state where several outbreaks of camel diseases including calves in many areas of the Somali region have been observed and become one of the major threats to camel production [12]. Understanding the major diseases of camel calves in the region with potential causes of mortality and setbacks in traditional camel rearing practices will fill the knowledge gaps about the epidemiology of camel calf diseases and thus would contribute to improved control and prevention measures. Therefore, this study was conducted with the objectives of identifying major camel calf health problems in the Somali region, determining the risk factors associated with the occurrence of the diseases, and assessing the current awareness of the pastoral community in the study area about the impact of diseases of camel calves.

## Materials and methods

### Study area

The study was conducted in three specific zones of the Somali region, namely Jarar, Qorahey, and Shabelle. These zones were chosen because they represent different agroecological areas where camels are reared. In each zone, two districts were purposefully selected based on criteria such as a high camel population, accessibility, and convenience. These districts include Degahbur, Birkot, Sheykosh, Kebridehar, Gode, and Adadle. The Somali Regional State (SRS) is located in the easternmost part of Ethiopia. Its capital city is Jigjiga. The SRS shares borders with the Ethiopian regions of Oromia, Afar, and Dire Dawa to the west. To the north, it borders Djibouti, while to the north, east, and south, it shares borders with Somalia. In the southwest, it is bordered by Kenya. The SRS has a total population of 4,445,219. With an estimated area of 279,252 square kilometres, the region has a population density of approximately 15.9 people per square kilometre [13]. In general, the Somali region experiences an arid and semi-arid climate. The average annual rainfall ranges from 200–700 mm/year. The region has two main rainy seasons, known locally as Gu (April to June) and Deyr (October to November). These rainy seasons are alternated by two dry seasons, locally known as Haggaa (July to September) and Jilaal (December to March).

### Study population

The study population comprised camel calves found in herds of six districts of four zones (Degahbur, Birkot, Sheykosh, Kebridehar, Gode and Adadle) of Somali region very popular for camel holding and pastoralism.

#### Study design and sampling

A cross-sectional study was conducted from May 2017 to March 2019. A participatory epidemiological appraisal was used to gain a rapid overview of the range of calf health problems and traditional management, a measure of the importance that people place on each of them, and to identify and prioritize economically important diseases in target zones.

A multistage random sampling approach was used to collect data for the study. Initially, 12 pastoral areas from six districts were judgmentally selected as study sites. Within each pastoral area, five camel holdings were randomly selected to participate in the survey. This resulted in a sample size of 120 camel calf holders for the study.

Owners of all selected holdings as well as a purposefully chosen sample (based on knowledge of calf husbandry and diseases) of 2 key informants and 10 focus group candidates were used for the participatory survey in each district.

#### Participatory epidemiological investigation

To generate relevant data and to exploit endogenous knowledge of the local community on calf management and diseases the commonly used participatory appraisal methods were fully utilized. Accordingly, multiple tools under informal interviews, visualization, ranking, and scoring methods were employed [14]. Additionally, focus group discussions (FDG) were used to assess major calf diseases and their impact on mortality rates in the study area. Similarly, professional observational checklists were employed to record data on clinical sick camel calves in six different districts.

#### Direct observation and sample collection

The evaluation of the clinical health status of calves ≤ 12 months old in selected camel holdings was achieved through a combination of historical analysis and physical examination. By matching local disease names with their corresponding clinical symptoms, the specific health issues affecting the calves were successfully identified.

Ticks were collected from different anatomical sites in the half-body region. The collected adult ticks were preserved in a properly labelled plastic container containing 70% ethanol and identified using a stereomicroscope [15].

Skin scraping was collected from suspected crust lesions with clean surgical blades and a visual examination with the help of a microscope was performed to diagnose and identify mite infestation [16].

Fecal samples were collected from calves suffering diarrhea using sterile gloves and then transferred to properly labelled universal bottles. These samples were then subjected to an extensive microbiological analysis in a laboratory to isolate and identify bacterial pathogens. This analysis included the use of standard biochemical tests to facilitate the identification process [17, 18].

#### Prioritization and identification of major calf diseases

Camel calf diseases of great relevance to this specific pastoral community were then prioritized based on the data generated from participatory appraisals. As a result, a specified number of calf diseases was defined as most important and rendered for investigation.

#### Data analysis

All data collected was subjected to statistical analysis using SPSS version 20. Descriptive statistics mean, frequency and bar charts were used to analyze data. The prevalence was assessed using descriptive statistics, and the association of potential risk factors was assessed using the chi-square (χ^2^) test. Agreement between different informant groups was assessed using Kendall’s coefficient of concordance (W).

### Ethics approval

Ethical approval for this study was obtained from the research ethics committee of Jigjiga University. The committee reviewed the research in accordance with the rules and regulations outlined in the Ethiopian animal disease investigation (Proclamation No. 267/2002) and granted approval with permit No. JJU/REC/019/2017. Verbal consent was also obtained from the animal owners.

## Results and discussion

### Participatory epidemiology on management practice

In all the interviewed key informants and camel owners from the study area confessed that camels were the most dominant species kept as livelihood, while small ruminants were second, followed by cattle. Similarly, most of the herders raised camels for milk; sales as a source of cash, social prestige like marriage, transport, and meat (see Table 1).

**Table 1:** Role of camel production contributions in the livelihood of the respondents.

Study District	Milk	Sales	Social	Transport	Meat	Total
Degahbur	11	6	2	0	1	20
Birkot	19	0	0	0	1	20
Sheykosh	18	0	0	2	0	20
Kebridehar	18	0	0	0	2	20
Gode	18	0	0	0	2	20
Adadle	16	0	0	0	2	20
Total	100(83.2)	8(6.7)	2(1.7)	2(1.7)	8(6.7)	120(100%)

*Social: means here different social uses of camels like payment as dowry, blood compensation, gifts or religious sacrifices

Herds are composed of all age categories, in which camels older than four years were the predominant portion of herds, while calves were the smallest part of the herd. As indicated in the following table (see Table 2).

**Table 2:** Average Herd sizes of respondents in the study area.

Variable (Months)	Mean ± Std. Dev	Min	Max
Calf(<1)	1.95 ±1.800327	1	11
Young (1–4)	7.025±6.617347	0	41
Older(>4)	9.458333±10.03766	0	75
Total (120)	18.43333±14.71507		

In the study area, pastoralism was found to be the predominant production system, accounting for 76.7% of the participants. Additionally, 23.3% of the study participants identified themselves as agro-pastoralists. It was observed that the majority of animals in the area had unrestricted access to grazing lands, with 95.8% of the peasants practicing common grazing patterns. However, a small proportion (4.2%) had private pastures for their animals. The study revealed that the quantity of fodder produced for the animals was relatively low, as only 13.2% of the herders admitted to cultivating forages. Furthermore, only 12.5% of the participants reported adding mineral salt supplements to their animals’ diets. These findings are detailed in Table 3.

**Table 3:** Common characteristics of the production systems in the area.

District	Pastoral		Agro-pastoral	Communal grazing	Private grazing	Forage production	Salt supplementation
Degahbur		4(3.4%)	16(13.3%)	16(13.2%)	4(3.4%)	14(11.6%)	10(8.3%)
Birkot		20(16.7%)	0(0%)	20(16.7%)	0(0%)	0	0(0%)
Sheykosh		12(10%)	8(6.7%)	20(16.7%)	0(0%)	0	2(1.7%)
Kebridehar		18(15%)	2(1.7%)	19(15.8)	1(0.8%)	1(0.8%)	0(0%)
Gode		19(15.8%)	1(0.8%)	20(16.7%)	0(0%)	1(0.8%)	1(0.8%)
Adadle		19(15.5%)	1(0.8%)	20(16.7%)	0(0%)	0	2(1.7%)
Total		92(76.7%)	28(23.3)	115(95.8%)	5(4.2%)	16(13.2)	15(12.5%)

As outlined in Table 4, the majority of respondents (95%) practiced providing colostrum to newly born calves through suckling. However, a notable portion (62.5%) reported implementing restrictive measures when allowing newborn calves to suckle colostrum, while the remaining 37.5% allowed unrestricted suckling of colostrum.

**Table 4:** Colostrum provision practice to neonate calves on the first day.

Study District	Allowed	Not allowed	Free access	Restrictive access	Suckling	Hand Feeding
Degahbur	18(15)	2(1.7)	11(9.2)	9(7.5)	16(13.3)	4(3.4)
Birkot	20(16.7)	0(0)	13(10.8)	7(5.8)	20(16.7)	0(0)
Sheykosh	19(15.8)	1(0.8)	2(1.7)	18(15)	19(15.8)	1(0.8)
Kebridehar	19(15.8)	1(0.8)	4(3.3)	16(13.3)	19(15.8)	1(0.8)
Gode	20(16.7)	0(0)	6(5)	14(11.7)	20(16.7)	0(0)
Adadle	18(15)	2(1.7)	9(7.5)	11(9.2)	20(16.7)	0(0)
Total	114(95%)	6(5)	45(37.5)	75(62.5)	114(95)	6(5)

According to Table 5, the majority of study participants (72.5%) keep young calves around the periphery of their houses during the daytime. In contrast, 27.5% integrate neonates directly into the main herds. For the nighttime, 54.2% of participants keep newborn calves with their dams in designated outdoor enclosures. Additionally, 21.7% keep calves with their dams without enclosure outdoors around the house, while 14.2% stated that they keep young calves indoors overnight.

**Table 5:** Calf keeping practices.

Period	Calf Keeping Methods	Frequency	Percent %
Day keeping	Around the house	87	72.5
	In field with herd	33	27.5
Night keeping	Kept without dam indoors	17	14.2
	Kept with dam outdoors around the house	26	21.7
	Kept with dam outdoors in special enclosure	65	54.2
	Kept in enclosure with other calves	12	10.0

According to Table 6, the respondents identified the occurrence of diseases as the primary obstacle to the optimal rearing of camel calves, with 61.7% of them reporting it as a major constraint. This was followed by water shortage, which was stated by 30% of the respondents, and food shortage, which was mentioned by 6.6% of respondents.

**Table 6:** Major constraints for camel calf rearing in pastoral areas.

Study district	Water scarcity	Food scarcity	Diseases	Poor housing	Total
Degahbur	11	0	9	0	20
Birkot	3	1	16	0	20
Shekosh	10	0	10	0	20
Kebridehar	5	4	10	1	20
Gode	0	2	17	1	20
Adadle	7	1	12	0	20
Total	36(30)	8(6.6)	74(61.7)	2(1.7)	120(100)

Table 7 illustrates that the highest percentage of participants, accounting for 42.5%, reported that their primary means of coping with common constraints in the study area was to move around in search of water and pasture for their camels. Additionally, other commonly employed strategies in the study area included purchasing feed for the livestock, dividing the herd into smaller fractions, or selling the livestock to minimize the risk of disease. However, a small number of respondents admitted that they do not undertake any specific action to mitigate risks, instead choosing to patiently observe how many of their camels survive.

**Table 7:** Means of coping with constraints by herders to minimize risks.

Study District	Moving	Feed Purchase	Herd splitting	Selling	Nothing	Total
Degahbur	3	10	5	2	0	20
Birkot	4	0	11	0	5	20
Sheykosh	16	2	0	0	2	20
Kebridehar	9	8	3	0	0	20
Gode	5	12	3	0	0	20
Adadle	14	1	3	0	2	20
Total	51(42.5)	33(27.5)	25(20.8)	2(1.7)	9(7.5)	120(100)

#### Focus group discussion on major calf diseases

Twelve focus group discussions were conducted in twelve different pastoral areas, with two discussions held in each district. Within each pastoral area, 10–12 key informants were chosen to participate as respondents in the focus group discussions (see Table 8).

**Table 8:** Summary of the focus group discussion respondents.

Zone	District	Pastoral Village	No of FGD	No of Men	No of Women
Jarar	Degahbur	Gedi arr	1	8	2
		Gerwo	1	9	3
	Birkot	Hoosale	1	7	4
		Eel Qur	1	9	1
Qorahey	Sheykosh	Qururaxle	1	8	2
		Wijiwaji	1	9	3
	Kebridehar	Dalad	1	7	4
		Eel aar	1	9	1
Shabelle	Gode	Gebi cas	1	8	2
		Hadhawe	1	9	3
	Adadle	Harisog	1	7	4
		Malkasalax	1	9	1

#### Simple ranking

Participants ranked the camel diseases based on their impact on calf mortality, with Camelpox receiving the highest mean score of 4.75±0.452, followed closely by Calf Scour with a mean score of 4.08±0.669. The informant groups displayed strong agreement in their rankings, as indicated by a high agreement score (W = 0.728, P = 0.000) (see Table 9).

**Table 9:** Mean score and five camel diseases by mortality determined by livestock keepers through Simple ranking exercises.

Diseases(n = 12)	Mean ± SD	Rank
Furuq (Camelpox)	4.75±0.452	1
Daab (Calf Scour)	4.08±0.669	2
Ajaro (Contagious ecthyma)	2.42±1.084	3
Shilin (Tick)	2.17±0.718	4
Pneumonic Diseases	1.58±0.793	5

W = 0.728 (P<0.000)

During the participatory appraisal, participants were asked to rank commonly prevalent diseases in calves that lead to sickness. The results showed that Calf Scours were ranked as the most prevalent disorder among calves, with a mean score of 3.67±1.23. Contagious ecthyma followed closely with a mean score of 3.50±1.09 (see Table 10). However, the agreement among the respondents in the participatory appraisal was slightly weak, indicated by a low agreement score (W = 0.189, P< 0.059).

**Table 10:** The mean score of five calf diseases based on their prevalence, as determined by livestock keepers using simple ranking exercises.

Diseases(n = 12)	Mean ±SD Score	Rank
Ajaro (Contagious ecthyma)	3.50±1.09	2
Shilin (Tick)	2.33±1.56	4
Daab (Calf Scour)	3.67±1.23	1
Cadho (Mange)	3.17±1.59	3
Furuq (camelpox)	2.17±0.94	5

W = 0.189 (P<0.059)

#### Proportional piling

Proportional piling techniques were conducted in twelve FGDs from the districts of Jarar, Qorahey and Shabelle zones. Using the technique participants were allowed to give relative score out of 100 counters and accordingly to rank six common camel calf diseases listed by key respondents during semi-structured interviews.

The findings showed that calf scour (Daab) was determined to be the most significant disease affecting calves, with a mean score of 29.3±7.59. This was followed by Contagious ecthyma and Camelpox, both with mean scores of 24.3±6.65. It is worth noting that there was strong agreement among the informant groups from the two districts in Jarar zone when it came to scoring and ranking the diseases, as evidenced by a high agreement score of W = 0.899 (P<0.003). This indicates a consensus among the participants regarding the importance of these diseases in the area (see Table 11).

**Table 11:** Mean score of six calf diseases as determined by Jarar zone livestock keepers through proportional piling exercises.

	Informant group and Score				Mean ± SD Score	Rank
Diseases	Gediarr	Gerwo	Hoosale	Eel-Qur		
Ajaro(Contagious ecthyma)	15	24	28	30	24.3±6.65	2
Dhaleco(CSN)	3	2	6	5	4.0±1.83	6
Daab(Scour)	35	19	28	35	29.3±7.59	1
Furuq(camelpox)	32	23	22	20	24.3±5.32	3
Cadho(Mange)	8	16	8	5	9.3±4.72	4
Shilin(Tick)	7	16	8	5	9.0±4.83	5

**W = 0.899 (p< 0.003)** (CNS = Contagious skin necrosis)

From Qorahey zone, Camelpox was the most important calf disease with a mean score of 26.75±5.852 followed by calf scour with a mean score of 25.25±2.872. Strong agreement (W = 0.881 (P< 0.003)) was observed among the informant groups of the two districts in Qorahey zone for disease scoring and ranking. However, results from Shabelle zone revealed that Calf scour (Daab) emerged as the primary concern among calf diseases, with a mean score of 27.25±5.909. It was closely followed by Contagious ecthyma and Camelpox, both with mean scores of 23.50±6.557 and 25.50±4.203, respectively. The participants from the two districts in Shabelle zone displayed a strong agreement in their disease scoring and ranking, as evidenced by a high agreement score of W = 0.874 (P<0.004). This indicates a consensus among the informants regarding the significance of these diseases in the area.

#### Local disease characterization by clinical signs

Matrix scoring was conducted in twelve focus group discussions (FGDs) in six districts (Jarar, Qorahey, and Shabelle). Each clinical sign or indicator had thirty counters allocated to it, and the respondents were asked to distribute the counters among the six diseases based on how strongly the clinical sign was associated with each disease. The results of the matrix scoring for common camel calf diseases in the study area can be found in Table 12. The analysis of the matrix scoring revealed a high level of agreement among the twelve informant groups for all disease signs (W = 0.934 to 1.000; P<0.000). According to the informants, diarrhea was strongly associated with calf scour, with a mean score of 30.00+0.000. On the other hand, skin and oral lesions were attributed to Camelpox, Contagious Ecthyma, and Mange, with mean scores of 10.75+452, 817.50+2.611, and 6.25+0.452, respectively.

**Table 12:** Mean scores for matrix scoring of major calf diseases.

Clinical sign(n)	W*	Disease and assigned score^a^, Mean±SD					
		Contagious ecthyma(Ajaro)	Camelpox(Furuq)	Mange (Cadho)	Calf scour (Daab)	Pneumonia (Dhugato)	Tick (Shilin)
Nasal Discharge(12)	W = 0.93*	17.17±3.35	12.50±2.91	0.17±0.39	0.0±0.0	0.00±.00	0.00±0.00
Oral Lesion (12)	W = 0.95*	17.50±2.61	12.25±2.59	0.08±.29	0.0±0.000	0.00±0.00	0.17±0.39
Skin Lesion(12)	W = 0.99*	7.75±452	10.75±45	6.25±0.45	0.0±0.00	0.00±0.00	4.92±1.32
Poor Growth(12)	W = 0.95*	5.00±0.00	7.08±1.31	2.33±1.37	5.33±0.78	2.50±0.901	7.75±0.45
Coughing(12)	W = 0.92*	0.00±0.00	1.42±0.90	0.00±0.00	0.0±0.00	28.50±0.91	0.00±0.00
Fever(12)	W = 0.93*	2.50±0.91	16.0±0.00	2.00±0.00	2.0±0.00	5.50±0.91	2.00±0.00
Diarrhea(12)	W = 1.0*	0.00±0.00	0.00±0.00	0.00±0.00	30.0±0.00	0.00±0.00	0.00±0.00
Salivation (12)	W = 0.95*	17.50±2.61	12.25±2.59	0.08±0.29	0.00±0.00	0.00±0.00	0.17±0.39
Death(12)	W = 0.96*	5.58±0.52	9.00±1.81	0.67±0.49	9.33±.99	2.00±0.00	3.42±1.31

n: The number of informant groups involved in the matrix scoring w

W: Kendal coefficient of concordance

(*- P< 0.000)

a: The higher the score, the more strongly pastoralists associated that clinical sign with the given disease

**Seasonal calendar.** The seasonal calendar for livestock diseases is summarized in Table 13. Very strong agreement was recorded among the twelve informant groups from the study area regarding the seasonal occurrence of diseases; such as camelpox, contagious ecthyma, Mange, calf scour, and ticks and seasonal pattern (W = 0.670–0.903). The incidence of camelpox was found to be high during the long rainy season (Gu) and the short rainy season (Dayr) with mean score of 12.42±1.165 and 10.08±0.515, respectively.

**Table 13:** Summarized mean scores for matrix scoring of six camel calf diseases and seasons of occurrence.

Season (n = 12)	W*	Camel disease and mean score					
		Furuq	Ajaro	Cadho	Daab	Dhugat	Shilin
Hagga	W = 0.89	5.0±0.74	14.83±0.6	5.3±0.62	1.3±0.65	5.0±0.0	3.7±1.44
Dayr	W = 0.90	10.1±.52	5.1±0.29	9.9±0.67	13.4±1.56	7.9±0.3	8.7±1.14
Jilal	W = 0.82	2.50±1.31	5.1±0.29	4.9±0.29	0.42±0.90	2.4±1.4	5.0±0.43
Gu’	W = 0.69	12.4±1.16	10.0±0.00	9.92±0.29	14.8±0.72	14.3±2.0	12.58±2.5

**n**: The number of informant groups involved in the matrix scoring

**w**; W: Kendal coefficient of concordance

(*: P< 0.000)

**a**: The higher the score, the more strongly pastoralists associated that clinical sign with the given disease

### Observational clinical records and laboratory investigations

#### Prevalence of mite infestation

As Table 14 indicates, of the 234 samples calves 83(35.5%) of them tested positive for *Sarcoptes scabie var cameli*. The study found that infestation levels did not significantly differ between male and female camel calves. Infestation was also not significantly affected by body condition. However, age and place of origin did have a significant impact. Medium-aged calves (5–8 months) had higher infection rates compared to older calves (9–12 months). In addition, the lowest prevalence of infestation was observed in Jarar (40.0%) and Shabelle (18.8%), significant with a (P< 0.002).

**Table 14:** Summary of the prevalence of mange mite and associated risk factors.

Variables	Risk factors	Number Examined	Number Positive	χ^2^	P-value
Sex	Male	114	38(44.7%)	0.86	0.35
	Female	120	47(55.3%)		
Age	1–29 days	33	4(4.7%)		
	1–4 months	81	24(28.2%)	19.1	0.000
	5–8 months	72	30(35.3%)		
	9–12 months	48	27(31.8%)		
Body condition	Good	33	10 (11.8%)		
	Medium	96	32 (37.6%)		
	Poor	68	25 (29.4%)	3.32	0.263
	Very Poor	37	18 (21.2%)		
Place of Origin	Jarar	78	34 (40.0%)	12.68	0.002
	Qorahey	78	35 (41.2%)		
	Shabelle	78	16 (18.8%)		
Total		234	85(36.3%)		

#### Prevalence of ticks in the study area

According to Table 15, the overall prevalence of tick infestation among the 234 camels examined was 36%. The proportion of tick infestation was relatively higher in calves aged 5–8 months (38.1%) and calves with very poor body conditions (47.6%). In contrast, animals with good body conditions and calves aged 1–29 days had lower infestation rates at 8.3% and 6% respectively. These differences were statistically significant with a P<0.05. However, the study found that the infestation did not significantly vary based on the sex of the camels or their place of origin, with P > 0.05 for both factors.

**Table 15:** The prevalence of tick infestation based on the age, sex, origin and body condition score of camels in selected districts of Somali region, Ethiopia.

Variable risk factors	Category level	No Examined	No Positive	χ^2^	P-value
Sex	Female	114	40(47.7%)	0.063	0.801
	Male	120	44(52.4%)		
Age	1–29 days	33	5(6%)	11.4	0.010
	1–4 months	81	25(29.8%)		
	5–8 months	72	32(38.1%)		
	9–12 months	48	22(26.2%)		
Body Condition	Good	33	7 (8.3%)	29.7	0.00
	Medium	96	20 (23.8%)		
	Poor	68	40 (47.6%)		
	Very Poor	37	17 (20.2%)		
Place of Origin	Jarar	78	35(41.7%)	5.4389	0.065
	Qorahey	78	28(33.3%)		
	Shabelle	78	21 (25%)		
Total		234	84 (36%)		

**Fig 1:**
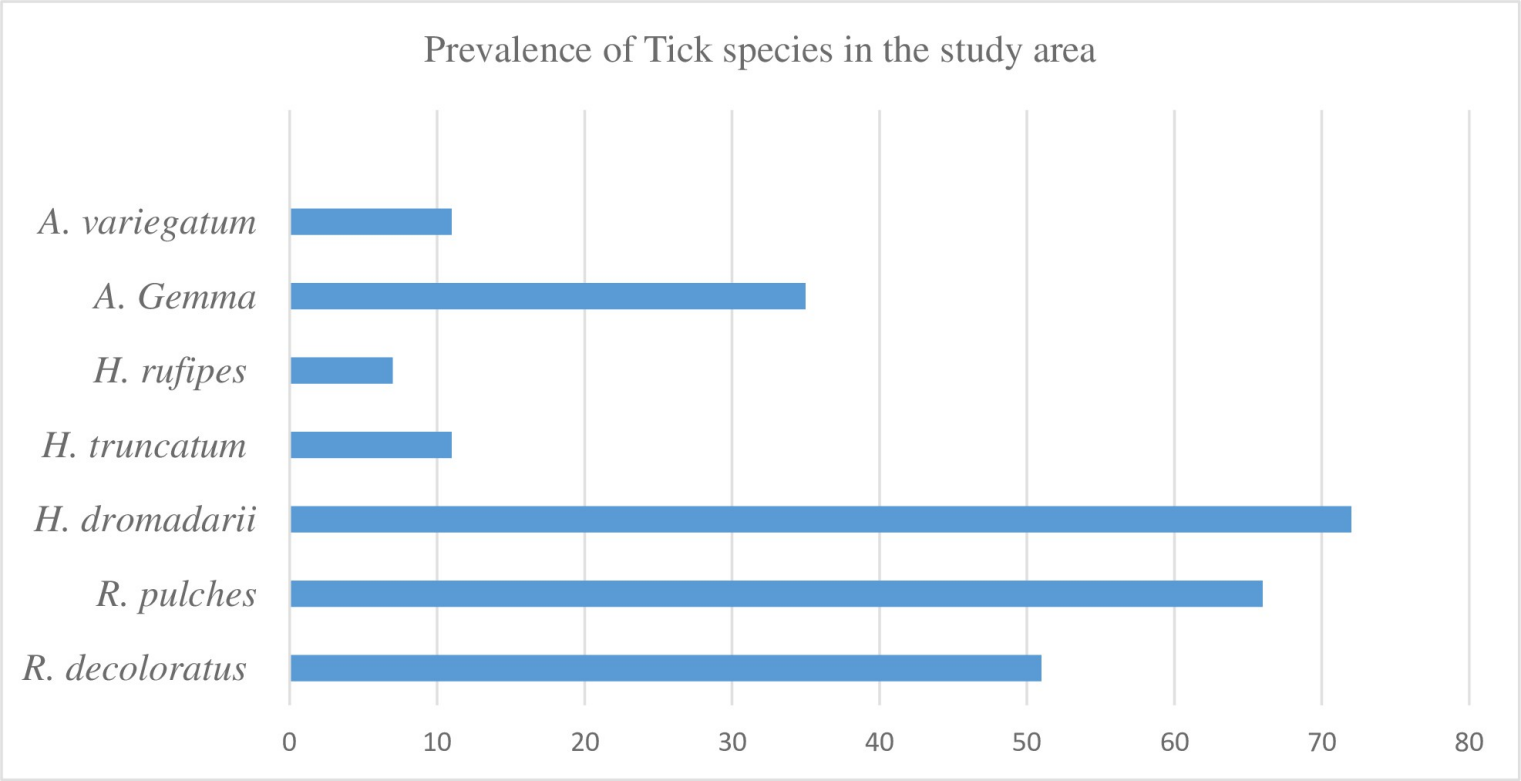
Distribution of camel ticks in the study area.

The study identified three species of *Hyalomma* ticks, with *H*. *dromadarii* being the most prevalent at 30.8%. *H*. *truncatum* and *H*. *rufipes* were less common, with prevalence rates of 4.7% and 3% respectively. Two species of *Rhipicephalus* ticks were also identified, with *R*. *decoloratus* accounting for 21.8% and *R*. *pulchellus* accounting for 28.2% of infestations. *A*. *Gemma* and *A*. *variegatum* were identified at prevalence rates of 15% and 4.7% respectively as indicated in Fig 1.

Out of the 84 camels infested with ticks, 26 (30.9%) were found to be simultaneously infested by three species, namely *R*. *decoloratus*, *R*. *pulchellus*, and *H*. *dromadarii*. Following closely, there were 21 (25%) camels that were infested with four species, namely *R*. *decoloratus*, *R*. *pulchellus*, *H*. *dromadarii*, and *A*. *gemma*.

### Prevalence of bacterial diarrhea among scouring calves

The result of the study revealed that out of a total of 234 camels inspected 74% (173/234) have diarrhea with relatively higher proportions 87(51.2%) and 69 (39.9%) from female animals and animals with medium body conditions respectively as summarized in the following Table 16.

**Table 16:** The prevalence of diarrhea based on the age, sex, origin, and body condition score of camels in selected districts of the Somali region, Ethiopia.

Variable (risk factors)	Category level	Number examined	Number positive	χ^2^	P-value
Sex	Male	114	86(49.7%)	0.262	0.609
	Female	120	87(50.3%)		
Age	1–29 days	33	28(16.2%)		
	1–4 months	81	58(35.5%)	2.403	0.493
	5–8 months	72	52(30.1%)		
	9–12 months	48	35(20.2%)		
	Good	33	20 (11.5%)		
Body condition	Medium	96	69 (39.9%)		
	Poor	68	52(30.1%)	6.505	0.089
	Very poor	37	32 (18.5%)		
	Jarar	80	59(34.1%)		
Place of Origin	Qorahey	80	57(32.9%)	0.177	0.915
	Shabelle	74	547(32.9%)		

#### Prevalence of *E*. *coli* and *Salmonella* among diarrheic calves

The overall prevalence of *E*. *coli* among diarrheic calves was 73.1% (171/234) and a slightly significant variation was observed in the prevalence of diarrhea among different age groups with a relatively higher proportion of 68 (39.8%) reported from animals with medium body condition. Similarly, the prevalence of *Salmonella* among diarrheic calves was 39.3% (92/234), however, there is no significant difference among animals with different body conditions. The overall prevalence of diarrhea was 74% among this about 34.6% was caused by *E*. *coli* while 38.9% were affected by *Salmonella* and *E*. *coli* (see Table 17).

**Table 17:** Evaluation of mean prevalence of *Salmonella* and *E*. *coli* among diarrheic calves in the study district.

Study District	No. of camels Examined	No. of infected calves with *Salmonella* and *E*. *coli*			χ^2^	P-value
		*E*. *coli*	*Salmonella*	*Salmonella* & *E*. *coli*		
Degahbuur	40	15(18.5%)	0	16(17.6%)		
Birkot	40	10(12.3%)	1(100%)	19(20.8%)		
Shekosh	40	14(17.3%)	0	14(15.4%)	7.92	0.92
Kebridaha	40	15(18.5%)	0	15(16.5%)		
Gode	40	15(18.5%)	0	15(16.5%)		
Adadle	34	12(14.8%)	0	12(13.2%)		
Total	234	81	1	91		

Among all the interviewed camel owners in pastoral villages, it was found that camels were the most common and important species kept for livelihood, followed by small ruminants and cattle. The primary reasons for raising camels were for milk production, selling them for cash, and for their social significance in events like marriage, transportation, and as a source of meat. These practices are in line with the traditional Somali pastoralism [19].

Pastoralism accounted for 76.7% of the dominant production system in the study area, while 23.3% of the study participants were agro-pastoralists. The vast majority of animals in the study area had the freedom to graze anywhere, as 95.8% of peasants practised common grazing patterns. The remaining 4.2% had private pastures for their animals. These findings differ from livestock production systems in the Fafan zone, where pastoralism accounted for 34.6%, agro-pastoralism for 50.0%, and sedentary farming for 15.8%. However, pastoralism emerged as the dominant system due to the arid and unpredictable fall rainfall in Jarar and Qorahey zones, making it the most favorable setting, as previously reported [3].

The study found that forage production for animals was low, with only 13.2% of herders admitting to practicing it. Similarly, only 12.5% of herders supplemented their animal’s food with mineral salts. It is important to note that since pastoralism is the predominant system practiced in the area, forage production and commercial salt supplements are not widely known or available. This lack of access to forage production resources and salt supplementation could explain the low rates observed in the study area. In pastoral zones of the Somali region of Ethiopia, forage production is not widely known or practiced due to traditional livestock management practices favoring free grazing over cultivating forage crops. The arid and unpredictable climate poses challenges, and limited access to resources like irrigation, agricultural inputs, and technical knowledge further hinders the adoption of forage production techniques. The nomadic lifestyle and extensive land use practices of pastoral communities are also not conducive to stationary forage cultivation. These factors collectively contribute to the limited knowledge and practice of forage production in the region [20].

Milk allowance to the calf is very critical, especially in the first three months of growth before the calf starts grazing [21, 22]. In this study, it was found that 95% of respondents allowed newly born calves to receive colostrum through suckling. However, only 62.5% of them allowed restricted access to colostrum, while 37.5% allowed unlimited access. The restrictions on suckling frequency and teat access likely reflect the competition between calves and humans (household/market) for milk. These early milk restrictions have significant negative implications for the survival and growth of camel calves. Some suggest that the increasing consumer demand for camel milk and stagnant productivity levels contribute to competition against calves, leading to high mortality rates in eastern Africa [4, 22].

According to the respondents, the occurrence of diseases is reported to be the main challenge in rearing camel calves, with 61.7% of respondents identifying it as a major constraint. This is followed by water shortage, reported by 30% of respondents, and food shortage, reported by 6.6% of respondents. These findings align with a previous study by [3].

The high prevalence and severity of diseases in the study area can be attributed to various factors. These include a lack of veterinary services, poor animal husbandry practices, and the practice of keeping a mixture of livestock species together. These conditions contribute to increased incidences of diseases among camel calves [23]. Additionally, the ecological nature of the study area, which is arid and semi-arid, plays a role in the prevalence of diseases. The region experiences erratic rainfall patterns, which makes it more susceptible to frequent droughts. These droughts are further exacerbated by global climate change, which has an impact on pasture and water availability in the region. The limited availability of pasture and water resources negatively affects the health and well-being of camel calves, making them more susceptible to diseases [24].

According to the findings, the majority of participants (42.5%) reported that their main coping strategy in dealing with common constraints in the study area is to move around in search of water and pasture for their camels. This highlights the nomadic nature of their livelihoods, as they rely on finding suitable resources for their livestock. Furthermore, other common coping strategies mentioned by participants include purchasing feed for their livestock, splitting the herd into smaller fractions, or selling the livestock to avoid the risk of losing them to disease. These strategies aim to mitigate the risks associated with limited resources and disease outbreaks. Interestingly, a few respondents mentioned that they do not actively take any specific actions to reduce risks but instead passively observe and let nature take its course. This might indicate a lack of alternative options or resources available to them. It is worth noting that similar coping activities have been reported in other pastoral areas of the country, suggesting that these strategies are commonly practiced among pastoral communities to address constraints and risks related to livestock rearing [4, 25].

Semi-structured interviews with livestock keepers revealed their ability to identify and describe the key fatal diseases affecting camel calves, as well as the most prevalent diseases in their area and the corresponding seasonality. The identified local names for these diseases included Furuq (camelpox), Ajaro (Contagious ecthyma), Daab (calf scour), Shilin (Ticks), and Dhugato (pneumonic diseases). Livestock herders not only provided detailed information on the affected organs and symptoms of these diseases but also demonstrated community awareness of their seasonal occurrence. Furthermore, the results of participatory epidemiology revealed that informants in the study area ranked Camelpox, contagious ecthyma, calf scour, ticks, and non-specific pneumonic diseases as the most frequent and deadly diseases affecting camel calves. Livestock herders not only described the affected organs and symptoms of these diseases but also demonstrated a deep understanding of the seasonality of disease occurrences. There was a strong consensus (W = 0.899, p < 0.003) among the informants from all focused group discussions regarding the seasonality of disease occurrences. This indicates that the community has a robust understanding of when these diseases are more likely to affect camel calves. The agreement observed between the informant groups and veterinary literature regarding the signs and symptoms of these important camel calf diseases highlights the valuable knowledge and expertise held by Somali pastoralists in symptomatic diagnosis of camel diseases. This finding supports the notion that the Somali pastoralists possess a wealth of knowledge and understanding in the field of camel healthcare, as indicated by [26–28]. This can be attributed to the long tradition of camel herding has enriched local knowledge of diseases through the ages, also frequent observation of diseases has increased disease familiarity among informants. Diseases affecting the integument system of calves such as mange, tick infestation, contagious ecthyma, pox, and contagious skin necrosis were quite common in the study area similar to other reports [29, 30].

The findings of the conventional study indicate that the prevalence of mange, ticks, and bacteria-induced diarrhea in the study area is 35.5%, 36%, and 74%, respectively. It is worth noting that the prevalence of diarrhea is particularly high, accounting for 74% of the total cases studied. Further analysis reveals that within this 74% prevalence, 46.8% of the cases can be attributed to *E*. *coli* infection, while 52.6% are associated with a combination of *Salmonella* and *E*. *coli*.

Calf diarrhea, as observed in the study, emerges as a complex condition with multiple potential causes. These include bacterial infections that manifest as yellowish, watery diarrhea with blood, as well as viral infections characterized by yellowish, watery feces. Furthermore, internal parasites, excessive milk consumption leading to white diarrhea, and sudden changes in feed are identified as additional contributing factors to this multi-causal background. It is crucial to acknowledge that the disease’s etiology is largely attributed to mixed infections involving various microbial agents, with *Salmonella* spp. and *E*. *coli* being the most notable among them [31, 32]. This suggests that the presence of multiple pathogens significantly contributes to the occurrence and prevalence of calf diarrhea. These findings emphasize the importance of understanding the various factors and agents involved in the development of calf diarrhea. A comprehensive approach is necessary to effectively manage and prevent this condition, taking into account both bacterial and viral infections, as well as parasites and dietary factors. Targeted interventions against *Salmonella* spp. and *E*. *coli* may prove to be particularly beneficial in reducing the prevalence of calf diarrhea in the study area [30].

## Conclusions

Camel production is vital for the livelihoods of pastoralists in Ethiopia’s semi-arid regions, especially in the Somali region. It provides income through milk and meat production, transportation, draft power, and social services such as dowry, blood compensation and prestige. However, the study reveals significant challenges due to diseases like camelpox, contagious ecthyma, calf scour, tick infestation, and nonspecific pneumonia. Additionally, poor management and husbandry practices such as restricted colostrum feeding, inadequate concentrate and salt supplementation, improper housing, and limited availability of feed and water contribute to the challenges identified. Recommendations include raising awareness about calf management, improving veterinary services, and conducting further research on camel diseases and calf immunity. Implementing these recommendations is expected to enhance camel health, productivity, and the well-being of pastoralist communities.

## Supporting information

S1 File (ZIP)
