# Supplementary material for: Participatory epidemiology on major camel calf health and management problems in pastoral and semi-pastoral zones of Somali region, Ethiopia
Source: PLoS One. 2024 Mar 29;19(3):e0301551. doi: 10.1371/journal.pone.0301551 (PMC10980207; doi:10.1371/journal.pone.0301551)
Supplement: S1 File — (ZIP) [file pone.0301551.s001.zip › Supporting Information/Qustionaires.docx]

**Jigjiga University**

**College of Veterinary Medicine**

**Research, Publication and Technology Transfer**


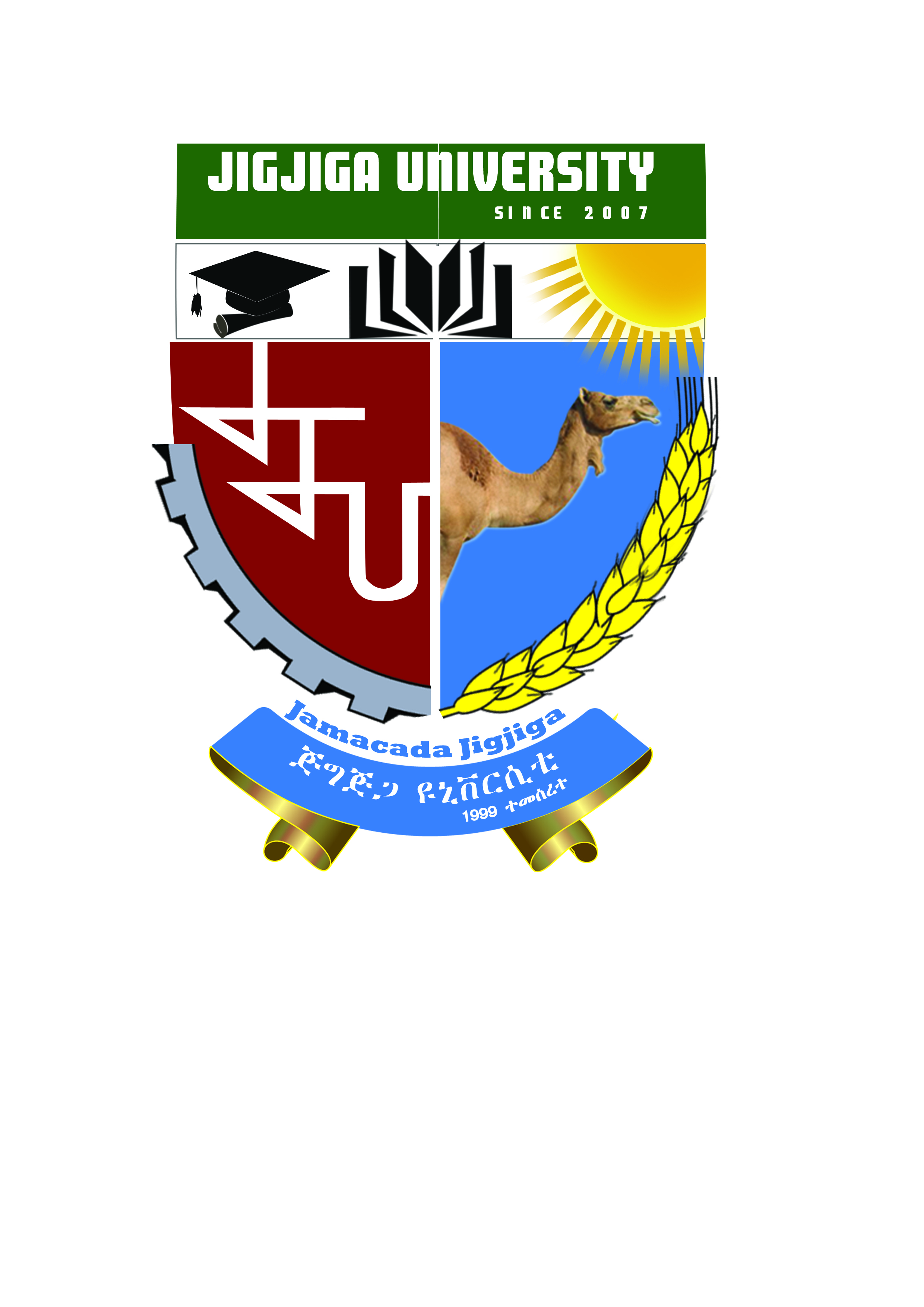


**Major Camel Calf Health Problems and Associated Risk Factors in Selected Zones of Ethiopian Somali Region**

**Semi-Structured Questionnaires, Focal Group Discussions and Participatory Epidemiology Appraisal**

**Date:____ _____ ________**

**I.** **General Information**

1.a) Zone: ______________ b). District: ___________ c). Pastoral Village_______

3. a) Age of Respondent_______ b) Sex of Respondent________ c) Level of Education ___

**II. Camel Production**

4. Production system_______________

5. What is the grazing land owning system in the area?

a). Communal grazing system ___ b). Individual grazing system ____

6.Types of livestock that are currently kept ?

1. _________
2. _________
3. _________
4. _________

7. a) No of Camels _____________ b) No of Camel Calves( < 1year )________ c) No of male calves_______ d) No of female calves____

| Total Camels | Mature females | Imature Heifers | Mature Bulls | Immature bulls | Female calves | Male calves |
| --- | --- | --- | --- | --- | --- | --- |
| No |  |  |  |  |  |  |
|  |  |  |  |  |  |  |

8.Do you give Colustrum to your calves in the first 1st day of life? a) Yes __b). No __

If colustrum is given, how do you provide it? A). Free access __B). With restriction __

If Colustrum is not given please specify the reason______________________

If colostrum is given with restriction give reason___________________

9. What is the first days colustrum feeding method. a) Suckling __b). Hand feeding __c) Both _

10. Volume of Colustrum fed per day in first 2 days a). <1 cup b) 1 cup c) 2 cup d) 3 cup

11. What is the source of feed for Camels and calves production? List in orders ____________________________________________________________________________________________________________________________________________________________12. Any feed supplements for calves and type ?

______________________________________________________________________________

13. Are there feed related problems for Camel rearing in your area? a). Yes __ b). No __

If yes what were the actions to be taken to overcome the problems?

A. Moving/Mobility B. Supplementation C. Herd Splitting D. Selling of Camels E. Others specify_____________

14. Do you practice production of improved forage for camel calves in the area?

A). Yes B). No. *If yes, which types of forages are you producing? ______________________________

15. What are the main problems for availability of food concentrates for your camel production?

A). Shortage of access B). Costly

C). Lack of awareness D). Combination of all E). Others________

16. What is the length of grazing/browsing time per day (in hours) for camel calves in the area?

A).1-6 hours B). 7-10 hours C). More than 10 hours

17. Watering intervals?

a). Every day b). Every two days

b). Every four days d). Every week

e). More than 15 days

18. Source of water?

a) River b). Stream

c). Pond d). Shallow well/open well

e). Deep pump well f).Others specify________________

19. What is the housing type of the calves at night?

1. kept with dam outdoors around house
2. kept with dam outdoors in special enclosure
3. graze away from home with other calves
4. kept in enclosure with other calves
5. Other (specify)________________

20. What are the major Camel Production problems in your area?

A. Feed shortage B. Water shortage C. Disease D. Housing E. Others (specify)__________

**III. Camel Calf Diseases**

21. What are the major camel calf diseases in your area? List in order of prevalence and importance?

Youg (before weaning i.e. start of green fooder or range grazing/browsing) calf health problems

| Prevalence | | Fafatlity (risk of mortality) | |
| --- | --- | --- | --- |
| Rank | Local disease names | Rank | Local disease names |
| 1^st^ |  | 1^st^ |  |
| 2^nd^ |  | 2^nd^ |  |
| 3^rd^ |  | 3^rd^ |  |
| 4^th^ |  | 4^th^ |  |
| 5^th^ |  | 5^th^ |  |
| 6^th^ |  | 6^th^ |  |
|  |  |  |  |

Weaned calf health problems

| Prevalence | | Fafatlity (risk of mortality) | |
| --- | --- | --- | --- |
| Rank | Local disease names | Rank | Local disease names |
| 1^st^ |  | 1^st^ |  |
| 2^nd^ |  | 2^nd^ |  |
| 3^rd^ |  | 3^rd^ |  |
| 4^th^ |  | 4^th^ |  |
| 5^th^ |  | 5^th^ |  |
| 6^th^ |  | 6^th^ |  |

22. List of common skin diseases? only calves

23. List of common GIT diseases? Diseases that cause diarrhea, vomiting, loss of appetite, abdominal distension ?

24. List of common Respiratory Diseases? Coughing, sneezing, nasal discharge, dyspnea orshallow breathing?

25. List of common Eye problem?

**IV. Retrospective Camel Calf Health Problem & Mortality Study (last year)**

**Number of calves born in the herd last year ________**

**Number of unweaned calves owned at start of last year ________**

**Number of weaned calves owned at start of last year _____________**

**Number of camel calves facing illness over the last year (Eg = 5)**

**Number of camel calves that died over the last year (Eg = 2)**

**Description of previous year health problem & mortality information**

| **Variables** | **Cases observed in herd last year** | **Calf information** | | **Case Description** | | |
| --- | --- | --- | --- | --- | --- | --- |
|  |  | **Sex** | **Age at Illness (months)** | **Symptoms** | **Duration** | **Treatment given** |
| **Health problems** | **1** |  |  |  |  |  |
|  | **2** |  |  |  |  |  |
|  | **3** |  |  |  |  |  |
|  | **4** |  |  |  |  |  |
|  | **5** |  |  |  |  |  |
| **Mortality** | **1** |  |  |  |  |  |
|  | **2** |  |  |  |  |  |

26. How many of your calves got sick last one year?________________________

27. List diseases in locale name that cause sickness in calves?

28. What do you think that cause these Diseases?

29. What kind of signs have you observed on sick in calves?

30. What was treatments given/ measurement taken to manage sick calves?

31. What was the outcome? a) No of dead_______ b). No of recovered______________

**Date:____ _____ ________**

**I.** **GENERAL INFORMATION**

1.a) Zone: ______________ b). District: ___________ c). Pastoral Village_______

2. No of Respodents______________

**II. FOCAL GROUP DISCUSSION**

3. What the major problems hindering the successful rearing of camel calves in your area?

- Calf mortality
- Feed or grazing shortage
- Camel calf diseases

4. What are the major causes of camel calf mortality? List in order of importance

5. In the past years were there disease outbreaks which severely affected camel calves?

6. Are camel calf diseases increasing or decreasing in your area in case of frequency and severity?

**III. PARTICPATORY EPIDEMIOLOG**

**1. Visualization**

Visualization, is the second important group of participatory methods, participatory mapping, seasonal calendars, proportional piling are examples of visualization methods

**1.1 . Participatory Mapping**

Draw key sites of the study area, Animal health post, livestock market, watering site, grazing area, roads, migration paths, neighboring kebeles.

**1.2.** **Timelines:**

- Draw a line on the ground or paper
- Think about the past 5-10 years, tell us about any major disease outbreaks or events in Camel calves – i.e. times when many camel calves were affected by a disease or died

**1.3. Seasonal calendar**: Write occurrence of camel calf diseases in respect to seasons?

| Season | |  | |  | |  | |  | |  | |  |
| --- | --- | --- | --- | --- | --- | --- | --- | --- | --- | --- | --- | --- |
| Rain | |  | |  | |  | |  | |  | |  |
| Av. Pasture | |  | |  | |  | |  | |  | |  |
| Dry | |  | |  | |  | |  | |  | |  |
| Dairhea | |  | |  | |  | |  | |  | |  |
| coughing | |  | |  | |  | |  | |  | |  |
| Skin diseases | |  | |  | |  | |  | |  | |  |
| death | |  | |  | |  | |  | |  | |  |
|  | |  | |  | |  | |  | |  | |  |
|  | |  | |  | |  | |  | |  | |  |
|  | |  | |  | |  | |  | |  | |  |
|  |  | |  | |  | |  | |  | |  | |
|  | |  | |  | |  | |  | |  | |  |
|  | |  | |  | |  | |  | |  | |  |

**3. Ranking and scoring**

Ranking and scoring methods were the third main group of participatory methods and usually required informants to compare different variables using either ranks or scores.

**3.1 Simple Ranking**

1. List in order diseases that cause greatest impact on camel calf health?

| Mortality | Morbidity | Prevalence | Incidence | Economy |
| --- | --- | --- | --- | --- |
|  |  |  |  |  |
|  |  |  |  |  |
|  |  |  |  |  |
|  |  |  |  |  |
|  |  |  |  |  |
|  |  |  |  |  |
|  |  |  |  |  |

**3.2. Pair wise ranking**

|  |  |  |  |  |  |
| --- | --- | --- | --- | --- | --- |
|  |  |  |  |  |  |
|  |  |  |  |  |  |
|  |  |  |  |  |  |
|  |  |  |  |  |  |
|  |  |  |  |  |  |
| Score |  |  |  |  |  |
| Rank |  |  |  |  |  |

**3.3. Proportional Piling**

Using counter allow herders to allocate proportion to sick calves in the herd in respect to mortality and morbidity

**4. Matrix Scoring**

|  |  |  |  |  |  |
| --- | --- | --- | --- | --- | --- |
| Death |  |  |  |  |  |
| Diarrhoea |  |  |  |  |  |
| Abortion |  |  |  |  |  |
| Nasal & ocular discharge |  |  |  |  |  |
| Mouth lesions |  |  |  |  |  |
| Skin lesions/nodules |  |  |  |  |  |
| Coughing/sneezing |  |  |  |  |  |
| Poor growth |  |  |  |  |  |
| Cost of treatment |  |  |  |  |  |

SEMI SRUCTURED QUESTIONAIRRE FOR ANIMAL HEALTH WORKERS

1. can you list the most common camel calf diseases around this area?

2. List in order of prevalence?

3. List in order of mortality?

4. List in order of difficult in treatment?

5.
